# The Rational Appropriateness of Group-Based Pride

**DOI:** 10.3389/fpsyg.2022.848644

**Published:** 2022-04-28

**Authors:** Mikko Salmela, Gavin Brent Sullivan

**Affiliations:** ^1^Practical Philosophy, Faculty of Social Sciences, University of Helsinki, Helsinki, Finland; ^2^Center for Subjectivity Research, Department of Communication, University of Copenhagen, Copenhagen, Denmark; ^3^International Psychoanalytic University Berlin, Berlin, Germany

**Keywords:** group-based pride, rational appropriateness, *we-mode*, *I-mode*, families, close relationships

## Abstract

This article seeks to analyze the conditions in which group-based pride is rationally appropriate. We first distinguish between the *shape* and *size* of an emotion. For the appropriate shape of group-based pride, we suggest two criteria: the distinction between *group-based pride* and *group-based hubris*, and between *we-mode* and *I-mode* sociality. While group-based hubris is inappropriate irrespective of its mode due to the arrogant, contemptuous, and other-derogating character of this emotion, group-based pride in the *we-mode* is appropriate in terms of shape if it is felt over an achievement to which the group members collectively committed themselves. For the same reason, members of *I-mode* groups can feel appropriately proud of the achievement of their group if they have collectively contributed to it. Instead, group-based pride by mere private identification with a successful group can be rationally appropriate if it manifests the person’s reduced-agency ideal and is also part of a coherent pattern of rationally interconnected emotions focused on the same ideal. Moreover, we suggest that pride in the success of one’s family member or a close friend is typically felt over the *rise of social status* that one group member’s success grants to the group. However, social status cannot be valued for its own sake as this undermines the values upon which social status is founded. Instead, direct or indirect causal contribution to the success of one’s child, friend, or student can warrant group-based pride, which may be justified on the basis of shared values without causal contribution as well. Finally, regarding the size of group-based pride, members of *we-mode* groups are warranted to experience and express more intense pride than members of *I-mode* groups. Moreover, the proper intensity of this emotion depends on the particular other(s) to whom the expression is directed. Finally, criteria of appropriate size don’t apply to shared group-based pride as sharing increases the intensity of emotion by default.

## Introduction

In their article “Pride, shame, and group identification” (2016), Alessandro Salice and Alba Montes Sánchez argue that people sometimes feel proud and ashamed of the actions of others whom they perceive to belong to the same group as themselves. The phenomenological accent of such emotions is either on oneself as the member of a group to which the pride- or shame-inducing other belongs, or on the other as one who belongs to the same group as the subject of emotion. The authors maintain that in both cases, the social self is the *target* (intentional object) of emotion whereas the other is its *focus*—a “background object having import to which the target is related in such a way as to make intelligible the target’s having the property defined by the formal object” (Helm, 2010, p. 58). Thus, when for instance parents feel proud of their daughter who has won a Nobel prize, the parents feel proud of their social selves because of their daughter’s achievement. Here the daughter’s achievement is a background object of the parents’ pride, whereas the parents themselves are the target of this emotion. Finally, the *formal object* or *core relational theme* of pride is “enhancement of one’s ego-identity by taking credit for a valued object or achievement, either of our own or that of someone or group with whom we identify” (Lazarus, 1991, p. 122). In feeling proud of their daughter, the parents experience enhancement of their ego-identity or self-esteem by taking credit for the daughter’s achievement with whom they identify as her parents.

While Salice and Sánchez Montes analyze the intentional and phenomenological structure of emotions felt on the basis of membership in social groups in a detailed manner, they do not discuss the rational appropriateness of such emotions. All they say is that a necessary yet not sufficient condition of appropriateness is objective membership in a group whose other members’ actions or achievements one feels proud of. We think that this topic deserves more thorough investigation as conventional emotion norms about the appropriateness of these emotions are not consistent. For instance, parents’ and grandparents’ pride in the achievements of their offspring is taken to be a warranted or even prescribed emotion in Western family ideology and probably is elsewhere in the world as well. Yet children’s shame of the drunken or criminal behavior of their parents is an emotion from which children should emancipate themselves, however, natural it is for them to feel ashamed of such behavior of close family members. Yet the need for emancipation suggests that this emotion is rationally inappropriate in the sense of *unfitting* (D’Arms and Jacobson, 2000) for the children of misbehaving parents. Or think about pride in the achievements of national sports teams—an appropriate emotion according to conventional feeling rules. Yet it seems that this emotion may become inappropriate if it motivates hooliganism or hostile behaviors against members of other groups. A question that emerges is whether only some expressions of group-based pride are inappropriate, or whether expressions of this kind should be understood in terms of a different emotion than group-based pride.

We believe that questions about the rational appropriateness of emotions felt on the basis of a group identification deserve a more thorough investigation with a wider range of examples and seek to initiate such investigation here (see also Salmela and Sullivan, 2016). By *rational* appropriateness we refer to the reasons that individuals have for feeling particular group-based emotions. These reasons can be evaluated in terms of their rationality and whether they render the emotion more or less warranted or justified. Rational reasons for feeling group-based emotions typically relate to the group members’ shared values, norms, concerns or goals—their “group ethos” in Tuomela’s (2013) terms—together with the members’ history of relationships, whereas irrational reasons—while ostensibly sometimes relating to the ethos of some group—typically go back to an individual person’s psychological needs for which a particular group-based emotion is felt and expressed.

Accordingly, the aim of this article is to contribute to empirically informed philosophical psychology. Our methodological approach is philosophical analysis in which we use the tools of conceptual analysis and normative argumentation as well as explore topics developed in theoretical social psychology and critical psychology. We focus on pride that in its paradigmatic forms can be felt either about one’s own actions and achievements or about those of others with whom we identify. Thus, Richard Lazarus defines pride as an “enhancement of one’s ego-identity by taking credit for a valued object or achievement, either of our own or that of someone or group with whom we identify” (Lazarus, 1991, p. 122). This definition of the core relational theme of pride involves group-based pride, and it is consistent with other accounts on the appraisal content of pride. Philosophers since Hume (1978) have argued that pride is felt about valued objects that the subject of emotion takes to be “connected” to the self. Taylor (1985) suggests that this connection is “belonging” in a broad sense. “[A] person who experiences pride believes that she stands in the relation of belonging to some object (person, deed, state) which she thinks desirable in some respect. This is the general description of the explanatory beliefs. It is because (in her view) this relation holds between her and the desirable object that she believes her worth to be increased, in the relevant respect. This belief is constitutive of the feeling of pride. The gap between the explanatory and identificatory beliefs is bridged by the belief that her connection to the thing in question is itself of value, or is an achievement of hers” (Taylor, 1985, p. 41). Objects of pride may include “an object or quality the subject possesses, an action she has performed, or the possession or achievement of a person to whom she is related, or of a group to which she belongs” (Taylor, 2013, p. 4078).

Before we examine the rational appropriateness of group-based pride, it is important to make two conceptual points. The first point concerns the term “group-based pride” that we apply as a general term to all instances of pride felt on the basis of a group identification. Thus, we do not distinguish between *vicarious pride* and *group-level pride* like the social psychologists Williams and Davies (2017) who state: “Vicarious pride arises in response to the success of a close other such as a family member, romantic partner or close friend. Group-level pride arises when a social group to which one belongs or with which one affiliates achieves a success (e.g., country or sports team).” (p. 43). We recognize that close relationships constitute groups that can be different in kind from more impersonal and larger groups to which we belong or with which we affiliate in terms of shared social identity or membership and a sense of belonging, and that these differences have certain implications for the appropriateness of pride felt in these contexts. Even so, since both vicarious and group-level pride are still instances of group-based pride, we do not see a reason for terminological promiscuity. Also, we prefer “group-based pride” to “hetero-induced pride” applied by Salice and Montes Sanchez as a more established term in interdisciplinary emotion research. The final qualification is that we understand group-based pride in the sense in which group-based emotions are generally studied in Intergroup Emotion Theory (Smith et al., 2007); that is, as an emotion that individuals feel on the basis of a group identification, yet typically without sharing these emotions with their fellow group members. Shared emotions have dissimilar properties to individual emotions irrespective of the emotion type (e.g., in terms of their typical intensity and the degree to which people feel free to be influenced by or share in them), and these properties are relevant to the appropriateness of shared emotions (see Salmela, 2014).

The second conceptual point concerns such verbal expressions as “I am proud of what you/we did.” It is important to observe that expressions of this kind do not always or perhaps even typically express an intention to take credit for the achievements of other persons and to feel proud of oneself. Instead, expressions of this kind may serve as recognition that highlights the value of the achievement to the relevant other(s), praising them for their achievement, rejoicing with them, and expressing group affiliation with them, as one of us has pointed out (Sullivan, 2007a,b, 2018). This observation emerging from Wittgensteinian philosophy of language and supported by qualitative interviews with people describing their group-based emotions has an important role in our discussion on the appropriate intensity of group-based pride below. Here we observe that rather than group-based pride, expressions such as “I am proud of what you/we did” may in situations of this kind express group-based happiness, which is a positive emotion about a success of one’s group or fellow group member that does not involve taking credit for the success in the first place (Sullivan, 2014).

## Criteria of Rational Appropriateness: Shape and Size; *We-Mode* and *I-Mode*

An important and influential distinction relating to the rational appropriateness of emotions concerns the *shape* and *size* of an emotion (D’Arms and Jacobson, 2000). An emotion is appropriate in terms of shape if its particular object has properties that render it an instance of the *formal object* or *core relational theme* of the emotion type, whereas it is appropriate in terms of size when the emotional response is neither too intense nor too mild, but of proper intensity, both in feeling and display. The notion of formal object refers to an evaluative property that each token emotion of the same type explicitly or implicitly ascribes to its particular object and that provides the standard of fittingness for individual emotions of that type (Kenny, 1963; De Sousa, 1987). Examples of formal objects are danger for fear, loss for sadness, and injustice for anger. Accordingly, a rationally appropriate emotion must be appropriate both in terms of its shape and size.^1^

How can we cash out these criteria of appropriateness for group-based pride? If we follow Lazarus, group-based pride is an emotion in which one enhances one’s ego-identity by taking credit for a valued object or achievement of another person or of a group with whom or which one identifies. An initial worry is whether we are warranted to take credit for valued objects or achievements of other people, whether or not this is psychologically possible. An agential view of pride that ties this emotion to personal achievements denies that group-based pride is ever warranted, or that it even counts as an instance of genuine pride as there is no agential connection to the relevant success (e.g., Kristjánsson, 2002; Fischer, 2017; Kauppinen, 2017). Instead, these cases have been suggested to manifest the phenomenon of *basking in reflected glory* or BIRGing (Cialdini et al., 1976) in which people hop on to winners’ bandwagon, expressing and publicizing their, however, thin relationship to successful others, in order to reap psychological benefits from a group identification with them.

Brady (2017) responds to this worry by arguing that the categorization of all instances of group-based pride as instances of BIRGing is not plausible. He points out that many cases in which we feel proud of the actions or achievements of others involve *reduced-agency ideals*: personal ideals that matter to us and in light of which we regard our lives as valuable, but which are satisfied and promoted by something other than *our* activity and efforts, such as organizations, teams, or other units with which we identify. This is not the case in BIRGing as contingent relations or encounters with successful others cannot plausibly be central to anyone’s identity. “Thus, someone can legitimately or appropriately feel pride in the success of their football team, when being a supporter of that team is one of their personal ideals, whereas someone else would be merely BIRG, or would be a “glory hunter,” if being a supporter of this team is not part of their identity or self-conception” (Brady, 2017, p. 19-20). For instance, being a fan of Liverpool FC may express support for the team’s iconic motto, “You’ll never walk alone” standing for peer support and solidarity—values that are plausible personal ideals. Instances of group-based pride that manifest reduced-agency ideals can then be a genuine form of pride. Yet it is not obvious whether these emotions are rationally appropriate—even according to Brady who supports their genuineness as emotions.

Brady offers a practical reason for doubting the appropriateness of group-based pride that is based on his social functionalist conception of pride. Following the social psychologists Tracy et al. (2010), Brady suggests that the experience and display of pride “helps individuals transform culturally valued achievements into higher social status,” whereas observers benefit by being able to “more effectively navigate the status hierarchy by showing appropriate deference, knowing whom to emulate, forming productive alliances, and facilitating their own status jockeying” (Brady, 2017, p. 21, 22). Therefore, individuals have a good *pro tanto* reason to regard an extended range of things as legitimate targets of pride, especially if they have little to feel proud about when it comes to their own agency. Given the need to maintain status and avoid rejection, there is a pressure on the subject to identify with a wider range of connections and associations that manifest achievements and purportedly valuable goods (such as being a customer of Sean Connery when he was a milkman in Edinburgh in his youth).

Yet there is a contrary normative pressure on the side of observers to restrict their judgments of merit so that they do not give respect, attention, and resources to those who do not deserve it. Brady describes this situation as a “normative arms race” between individuals who wish to expand the category of legitimate objects of pride for reasons of self-esteem and enhanced social status, and observers who wish to restrict this expansion for prudential reasons of their own. In this situation, agency emerges as a *pragmatic* criterion for regarding reduced-agency pride as genuine, yet still less appropriate in comparison to pride based on personal achievements. An agential account offers a more suitable measure of success and merit than reduced-agency ideals whose genuineness it is difficult to evaluate.

We agree with Brady’s conclusion about the genuineness of reduced-agency pride and its normatively weaker claim to appropriateness in comparison to agential pride. However, our reasons for agreeing with Brady are somewhat different. Their elaboration leads us to another conceptual distinction that we take to be central to the rational appropriateness of group-based pride. This is the distinction between *we-mode* and *I-mode* collectivity and groups emerging from Tuomela’s (2013) philosophical work.

A major distinction concerning types of collectivity in contemporary philosophy of collective intentionality is that between *I-mode* and *we-mode* collectivity (Tuomela, 2013). Tuomela argues that individuals may function in group contexts in either *I-mode* or *we-mode*. When they act or express attitudes in the *I-mode*, they function as group members, but their commitment to the relevant actions or attitudes is private, in other words, it is based on their goals as private persons. For instance, two individuals have the same destination and therefore decide to share the same taxi instead of each taking taxis of their own. The *I-mode* is the weakest form of collectivity; “it simply requires that the agents have an attitude with the same content and mutually believe that they have it” (Tuomela, 2013, p. 6). The *we-mode* is stronger. It requires that individuals intend to act together or have attitudes as a group for authoritative group reasons, and that they conceive of themselves as group members who are bound by and committed to what is collectively accepted and subject to collective commitment in the group. An example of a *we-mode* group is a team whose individual members have collectively committed to the same goal which they pursue together for the group reason grounded in their shared goal. Reaching the shared goal together provides the group members an authoritative group reason for feeling group-based pride about the joint achievement.

Between the *plain I-mode* and the *full-blown we-mode*, there is a type of collectivity that Tuomela refers to as either *pro-group I-mode* or *weak we-mode*. It resembles full-blown *we-mode* in the sense that individuals in this mode of collectivity have shared goals, such as going to see a movie or have lunch together, that they pursue together as a group. We come to have shared goals of this type because we believe that other members of our group have them, where this belief is either a *reason* or a *cause* or both for our adopting the same goal. The commitment to the goal in *weak we-mode* is private, but the goal is shared with others, unlike in the *plain I-mode* where the goals too are private. Nevertheless, *weak we-mode* collectivity resembles *plain I-mode* collectivity in being based on private and therefore possibly divergent reasons that are grounded in a personal rather than a collective commitment to the shared goal of the group members.^2^

We will suggest that it makes an important difference to the rational appropriateness of group-based pride whether the group as a member of which this emotion is felt is a *we-mode* group or an *I-mode* group, that is, whether the members’ commitment to the *group ethos*—certain shared beliefs, values, norms, concerns, etc. — is either collective or individual. The group members with a certain ethos come to have a shared social identity defined in terms of elements from the group ethos. However, the distinction between *I-mode* and *we-mode* becomes relevant only after the first criterion of appropriate shape. For if an instance of group-based pride does not qualify as appropriate in terms of its shape by being derogatory and aggressive against an outgroup, it does not matter whether it is felt in the *I-mode* or in the w*e-mode*. It is only when the first set of criteria is met that the second distinction becomes relevant. This kind of *lexical order* between criteria is familiar from John Rawls’s (1971) argument on the relationship of his two principles of justice.^3^ We suggest that a similar lexical order applies here as well. In the next two sections, we will first introduce a distinction between group-based pride and group-based hubris as the first standard of appropriate shape, followed by a distinction between we-mode and I-mode as the second standard of appropriate shape.

## The First Criterion of Appropriate Shape: Group-Based Pride vs. Group-Based Hubris

There is an influential and much studied distinction between two forms of pride, *authentic* and *hubristic*, in the literature on individual pride (Tracy and Robins, 2004, 2014; Tracy et al., 2010). The distinction concerns the attribution of personal success to different aspects of the self: to unstable and controllable aspects of the self such as one’s efforts in authentic pride, and to stable and global aspects of the self such as one’s abilities or identity in hubristic pride. Moreover, authentic pride is found to be associated with stable self-esteem, confidence, productivity, and prosocial behavior, whereas hubristic pride is associated with contingent self-esteem, low perceived social support, narcissistic self-aggrandizement and antisocial behaviors that are argued to function as defenses against repressed shame.

It is not obvious whether the distinction between authentic and hubristic pride is applicable to group-based pride. Insofar as group-based pride is felt about the achievements of one’s group, it does not seem relevant to distinguish between different types of this emotion on the basis of whether the achievement is attributed to the efforts or abilities of the group members. Insofar as the group has achieved something of value from the group members’ intentional perspective, group-based pride seems appropriate in terms of shape. Nor does it make much sense to call this kind of group-pride “authentic”; it is, rather, group-based pride period. What seems more relevant for appropriateness is the way in which group-based pride is felt about particular achievements of one’s group and expressed in a manner that involves humility and consideration of the other or others. Group-based hubris is a significantly different kind of emotion.

In group-based hubris, an achievement of one’s group has a merely causal and contingent role as evidence for the group’s greatness and superiority which is the constitutive appraisal of this emotion (Sullivan and Hollway, 2014). Thus, group-based hubris manifests as overconfidence in continued success in organizations, corporations, or sports teams. Besides appraisals of the group’s greatness and superiority, group-based hubris involves expressions that differ in *kind* from group-based pride in being arrogant and antagonistic toward competitors and other group(s) that are perceived as denying or doubting or challenging the privileged status of the in-group (Sullivan, 2017). In this understanding of group-based hubris, beliefs of the group’s greatness or superiority, together with expressions of arrogant boasting and negative group-based emotions such as group-based contempt and group-based anger with underlying shame, either personal or group-based, *replace* rather than *extend* group-based pride, which is a positive celebratory emotion about particular achievements of one’s group (Sullivan and Hollway, 2014; Sullivan and Day, 2019). The sense that one’s group is believed and felt to be special in group-based hubris is captured better by the notion of *collective narcissism* (Golec de Zavala et al., 2009) than by group-based pride even if group-based hubris may have contingent connections with experiences of group-based pride. Thus, group-based pride experienced alone or with others (e.g., during national celebrations, commemorations etc.) may turn into group-based hubris if an appraisal that one’s group should be or is entitled to maintain certain standards (i.e., to continue winning) or status (e.g., as the most powerful group), is special (i.e., has unique characteristics in contrast to other groups) becomes central in the emotion that is contingently reinforced by further group achievements. Group-based hubris can lead to anger, shame and misery when the status of the group cannot be sustained or appears to have been permanently lost (e.g., as in the loss of empire in the case of Britain which increases the appeal of reactionary political movements; Sullivan, 2021).

Since we argue that group-based hubris is a distinct emotion (or set of emotions and beliefs including collective narcissism) from group-based pride, we argue that group-based hubris, understood as arrogant, contemptuous, and other-derogating group-based emotion is inappropriate irrespective of its mode (*we-mod*e or *I-mode*). Group-based hubris is particularly skewed in the *I-mode* where an individual’s affiliation with a successful group (e.g., through being proud of a group representative) is based on a mere personal identification. Even if the group stands for values that qualify as reduced-agency ideals for the subject, pride in the group’s success does not warrant arrogance and derogation of other groups (e.g., to make a claim that New Zealanders world-beating COVID-19 response reflects their fundamental superiority to the members of all other nations in other matters as well). Yet such behavior can sometimes be seen among sports fans where the fans of the winning team mock and humiliate the fans of the losing team during their celebrations. Sharing group-based emotions with fellow group members feeds and reinforces this antagonistic dynamic that is less common when individuals experience group-based pride alone (e.g., when watching a live stream by oneself). Yet savoring the opponent’s defeat can be the flipside of group-based pride even in these situations, as every Finn who felt proud when Finland won World Championship in ice-hockey 1995 and 2011—both times after beating the arch rival Sweden in the final—knows. If excessive, such savoring of the outgroup’s defeat turns *I-mode* group pride into forms that are not only excessive and unjustified but also potentially hubristic if generalized superiority and overconfidence with arrogance and contempt toward the other come to replace a positive celebration of a particular achievement.

*We-mode* group-based pride is compatible with a more intense experience and expression of this emotion as membership in the group whose success is the object of pride is based on a collective commitment of the members to the group ethos, which makes the achievement “theirs” whether or not the particular member experiencing this emotion has participated in the successful joint action. However, this context does not warrant arrogant, contemptful, and outgroup-derogating group-based hubris either, and it is important to prevent the potential overgeneralization of group-based pride in an achievement into a subsequent desire, for example, to dominate other groups. In team sports, this usually is no problem as the players of opposing teams respect each other, and as an expression of this respect, they shake hands after the game. It is also rare to see members of the winning team celebrate their win in an arrogant, contemptuous, and boasting manner; it is more often the fans of the winning team who engage in this kind of inappropriate expression of their emotion if there is an established rivalry between the opposing teams, such as Liverpool FC and Everton, or Manchester United and Manchester City in football, for instance.

## The Second Criterion of Appropriate Shape: *We-Mode* and *I-Mode*

Now, when we have distinguished group-based pride from categorically inappropriate group-based hubris, it is time to bring back the distinction between *we-mode* and *I-mode* collectivity and to show what kind of analytic work we can do with it. The *we-mode* is more straightforward in this respect, for here the achievements of the group are by default shared in the sense of being distributed among group members rather than attributed to individual persons. For instance, when people feel proud of the achievements of their fellow group members, the emotion can be felt as sharing in *our* pride of something that we did together, provided that the achievement was a joint effort with similar or even importantly dissimilar individual contributions. There can be operative group members who are responsible for the execution of the group’s joint action whereas other group members have participated in the collective acceptance of the group goal on which the group’s action is rationally founded. Yet both kinds of group members can appropriately feel proud of the group’s achievement. Here the self is involved in the “we” whose members are collectively committed to achieving the shared goal, and therefore every group member can take credit and feel proud of the achievement. The collective intentionality framework incorporates how “the group constitutes the social identity of each individual “we-moder” (Tuomela, 2013, p. 24) but focuses also on three features that are not present in the same way as in collective intentionality approaches in the concepts and models of social identity theories: group reasons, collectivity (i.e., acceptance that group members have the same interests rather merely perceive themselves as being in the same boat), and collective commitment to the satisfaction of the group’s goals (Sullivan, 2018).

In *I-mode* groups, the group ethos is not shared in the same way as in *we-mode* groups. Instead of the members’ collective commitment to the group ethos, each member of an *I-mode* group has privately committed to the ethos. Yet individual members of *I-mode* groups can feel proud in the same way as members of *we-mode* groups insofar as they have contributed to the group’s achievement in one way or another. We can think about a homeowners’ organization to which individual homeowners belong out of private self-interest. When the members agree on submitting a complaint to the city council about its plan to build a major shopping mall to the neighborhood that would significantly increase traffic, pollution, and noise in the area, all members who support the complaint can feel appropriately proud if it turns out to be successful.

On the other hand, there are cases in which individuals take pride in the achievements of others with whom they affiliate by a private *I-mode* group identification, without contributing to those achievements in any way. Salice and Montes Sánchez (2016) suggest that an affiliation with a group makes the other group members’ achievement an intelligible background object for feeling proud of one’s social self. Another way of formulating this position would be saying that the group members’ achievement provides individuals who affiliate with the group a *reason* for feeling proud of their social identity as members of the group. However, pride in a social identity, however, valued by the subject, does not appear to be an appropriate object of pride in the first place.

This may appear to be a counterintuitive claim given that sexual minorities celebrate their identities on annual pride events all over the world. However, this pride must be seen in the context of a history of repression and humiliation that members of these minorities have experienced and in light of which their pride—even if it is ostensibly felt about identity—is also about the ongoing struggle for emancipation from repression, which is a collective achievement of sexual minorities that calls for a proud celebration (Salmela, 2014). This example shows that pride about a social identity is rarely just about the identity but about some value that the group stands for and promotes in its activities (often against a historical background of intergroup relations). Insofar as an achievement of the group depends on its constant maintenance, such as struggle against heteronormativity and its various remnants in contemporary societies, it may be possible to participate in this collective action and feel proud of doing one ìs part in a joint effort, which is a perfectly warranted object of group-based pride.

The situation changes if the individual who privately identifies with a group does not personally participate in the achievements of the group that he or she nevertheless values. Here we have the case of reduced-agency ideals that allows individuals to feel proud of the actions of others who promote those values. An example could be being proud of the comments of the Mayor of Bristol in supporting people who pulled down the statue of a historical figure involved in the slave trade following the 2020 Black Lives Matter protests. We agree with Brady (2017) that these cases may count as genuine and even appropriate instances of group-based pride. We suggest, however, that in order to qualify as rationally appropriate, group-based pride based on a reduced-agency ideal should be part of a wider coherent pattern of rationally interconnected emotions, both group-based and individual, of the person such that the person would feel fear when the ideal is endangered, anger against those threatening or violating against the ideal, relief when the threat to the ideal recedes, and so on (Helm, 2001). A coherent rational pattern of actual and counterfactual emotions of this kind, together with other ways of displaying group membership, can provide evidence that the person cares for the relevant reduced-agency ideal and is not merely a BIRGing fan of successful or otherwise prestigious groups.

What are the implications of these considerations for the group-based pride of sports fans about the achievements of their favorite teams? If we think about fans who have supported their favorite team through thick and thin throughout the entire season, such fans have certainly contributed to the success of their favorite team through their loyal support. Therefore, such fans may take *some* credit for the team’s success in a way that is analogous to the members of the team who operate in *we-mode* and therefore feel appropriately proud of the team’s success. Indeed, fans can form *we-mode* groups in which the members collectively commit themselves to supporting their favorite team throughout the season, which is a somewhat different goal than the team’s goal to win games and titles. These goals are not overlapping, but their conditions of satisfaction converge to the extent in which the fans’ group goal is satisfied when the team wins its games, which is also the condition of satisfaction of the team’s goal. The members of such we-mode fan groups can feel appropriate group-based pride when they succeed in supporting their favorite team (e.g., audibly in stadiums against rival supporters), thereby helping it to win its games.

In the case of national sports teams, the fans and the players are members of the same national group (at least in terms of citizenship) and the fans need no separate identification with their nation in order to experience group-based pride about the successes of their national team. Even so, individual fans of national sports teams resemble *I-mode* group members in their relation to the teams and their players who are the agents whose successes and failures are at stake. The teams operate in the *we-mode*, and success is their goal to which the team members have collectively committed themselves. The individual fans hope that their national team succeeds, but they cannot be parties in the collective commitment of the team with this content, nor can many of them contribute to the team’s success through their support in a tangible manner in major international tournaments such as a FIFA World Cup which are almost always played abroad. For these reasons, the fans’ relationship to the national team, which is mediated through the shared identity of a common nationality, resembles that of *I-mode* group identification. This means that the fans of national teams cannot take credit for the achievements of those teams in a literal sense even though those achievements provide fans with a reason for feeling group-based pride which can be both genuine and appropriate. Even so, the intentional structure of the fans’ emotion is somewhat different from that of the players whose group-based pride is in the *we-mode*. Thus, when the players express their emotion by saying that “*We* are proud that *we* won today,” both “we’s” in this sentence refer to the same we, that is, the players as a team. Whereas if the fans express their emotion with the same sentence, the first “we” refers to the particular fans who are speaking, while the second “we” refers to the team, and ultimately to the nation as the team which qualifies as *ours* by virtue of common national identity between the fans and the players. The fact that the group-based pride of the fans is in the *I-mode* means that they are warranted to *prima facie* less intense experience and expression of their emotion than the players whose group-based pride is in the *we-mode*. However, since the fans are also justified to celebrate and rejoice in the success of their national team, the mixture of these joyful emotions with group-based pride may result in an intensely felt and expressed positive emotion that is rationally appropriate to the fans. Indeed, this is what we see happening in spectator stands at stadiums and in public viewing locations where fans intensely celebrate the goals and wins of their favorite teams. However, intense celebration should not lead the fans into overgeneralizing the particular achievements of their favorite team into a belief of superiority in other unrelated group matters as this would transform the profile and valence of their group-based pride into group-based hubris.

## Appropriateness in Terms of Shape in Families and Other Close Relationships

A problem with the distinction of *I-mode* and *we-mode* is that it is difficult to apply it to some of our most important groups, that is friendships and families, that cannot be understood as either *I-mode* or *we-mode* groups in a straightforward sense. On the one hand, families and friendships appear to be normatively weaker than *we-mode* groups as they need not have a constitutive group ethos to which the group members have collectively committed themselves. The parents in a family may have shared values, but the children may not have been able to freely commit themselves to the values in accordance to which they have been raised, and therefore, the requirements of a *we-mode* group are not met. Moreover, even if family members could have some shared values that are understood to be constitutive of the family, parents and children often have personal values and projects that reflect the family members’ shared values only contingently. Therefore, children’s achievements need not reflect the attainment of some shared value of the members as in *we-mode* groups. Rather, children have their own goals, and parents feel proud when their children achieve those goals, whatever they are.

On the other hand, families and close relationships appear both normatively and affectively stronger than *I-mode* groups where the commitment of the members to the group ethos is private. It seems that family members and close friends can be *socially committed* to each other without a mediating group ethos to such an extent that the social commitment normatively and functionally replaces a collective commitment to a group ethos, giving rise to normative duties of care, protection, and support. Close relationships are also affectively more intimate than *I-mode* groups, and similarly to social commitments among group members, it seems that the reciprocal affective ties of love, loyalty, and solidarity between family members and friends need not be mediated through a group ethos which in *we-mode* groups grounds social commitments and felt intimacy between the members. Indeed, intimacy can be experienced even in a crowd of strangers with a shared social identity where it can associate with similar normative expectations of protection and support as in close relationships (Neville and Reicher, 2011), although it is not clear how reciprocal and enduring these expectations are among strangers, especially if they do not organize into a more permanent group. Yet these reciprocal affective ties are important as they give rise to a coherent pattern of rationally related emotions among family members and close friends when the well-being of one member is affected favorably or adversely in specific ways (Helm, 2010). Thus, for instance, other family members are frightened if one member falls ill, hopeful if doctors find a cure to the illness, relieved if the ill family member begins to get better, devastated if the illness turns out to be terminal, and so on. Importantly, a failure to experience the relevant emotions in the appropriate situations is not merely a psychological failure but also a normative failure, because there is a rational ought to feel these emotions that associate with the family membership.

The first consideration toward understanding the appropriateness of group-based pride in families and close relationships and family-like groups is to explore to what extent group-based pride is an empathetic response to the pride of other people. We understand empathy, following Peter Goldie, as “a process or procedure by which a person centrally imagines the narrative (the thoughts, feelings and emotions) of another person” (Goldie, 2000, p. 195), using perceptions of other people’s emotional expressions as cues in invoking a fellow-feeling with them. We readily empathize with our significant others and come to share the emotions that we perceive or imagine them feeling. Thus, the parents’ pride can be an empathetic response to the pride that they perceive or presume their daughter feeling about her winning a Nobel prize. In analogous manner, parents’ pride in a developmental milestone of their infant child could be their empathetic response to the child’s achievement that the child him- or herself is not capable of fully grasping as such because the child has no understanding of the importance of, for example, saying one’s first words, or taking one’s first steps. The child may feel and express joy and surprise at his or her newly found skills even before the child is capable of feeling proud of these achievements, which requires having the concept of the self (e.g., Hart and Matsuba, 2007). Here, the parents’ pride could be understood as being felt vicariously *from a perspective in which the child was able to understand the importance of those achievements* (Williams and Davies, 2017). Accordingly, group-based pride of this kind could be an empathetic response to actual or imagined pride of another person that either is or *would be* appropriate from the perspective of the person. An empathetic pride of this kind is not limited to infancy as it can be felt at other times as well.

Importantly, if group-based pride in families and close relationships and family-like groups is an empathetic response to the actual or imagined appropriate pride of another person, this emotion should not be interpreted as a group-based pride of the social self, except in a psychological sense, because the proper subject of the emotion is the other person (or persons, such as this family). However, this interpretation of parental pride seems unlikely in many cases, because the phenomenological accent of these emotions—to use Salice and Montes Sánchez (2016) useful expression—is on the parents themselves rather than on the child whose achievement provides a reason for it. Boasting of the achievements of significant others betrays a different kind of emotional attitude than merely feeling proud *with* or *on behalf of* the other person or persons. Instead, it suggests an absence of genuine care or interest in the other, or a psychological inclusion of the other in the self (potentially a kind of identity fusion also), with the consequence that group-based pride becomes mixed with personal pride of one’s involvement or contribution to the success of the other. More generally, it seems that we need not engage in an empathetic understanding of the emotion-eliciting situation from another person’s perspective in order to feel proud of his or her actions. This suggests that group-based pride about the achievements of significant others cannot be generally understood as an empathetic response on behalf of those others.

Second, a causal contribution to the success of another person may be important for the rational appropriateness of group-based pride even if it does not figure in the phenomenology of these emotions, as Salice and Montes Sánchez (2016) point out. The example of proud parents is a case in point. It may be *prima facie* appropriate in cases where the parents with their caring and loving parenting have indirectly yet importantly contributed to the success of their offspring. Accordingly, foster parents can feel appropriately proud of the success of their adopted children, whereas there is something morally repulsive in the pride of biological parents in the achievements of children who have been taken into custody and raised by others due to the parents’ neglectful or abusive behavior. Another, more invisible type of abuse is involved in parenting with achievement-oriented psychological control. This kind of parenting has been associated with rising levels of stress and anxiety, and reduced well-being, academic performance, and social skills among adolescents (e.g., Wang et al., 2007; Oudekerk et al., 2015; Cucillo et al., 2017). Even if the children of psychologically controlling parents sometimes succeed, the price for the children may be too high. Moreover, there is evidence that psychologically controlling parents are particularly prone to feel proud of the achievements of their children as these parents often see their children’s achievements as surrogates for their own unfulfilled ambitions (Brummelman et al., 2013; Wuyts et al., 2015). This kind of instrumental attitude toward one’s children violates their dignity as persons who are ends in themselves, to use Kant’s expression.^4^

The social connectedness of family members and close friends that takes the form of social commitments with normative duties of care, protection and support as well as affective intimacy in the form of love, loyalty, and solidarity means that (core) families and close friendships are capable of constituting strong forms of “we” whose individual members psychologically include other group members into their selves. We are mothers or fathers of particular children, or someone’s daughters or sons or friends even if we don’t have children, and stripping us from these vital identities and treating us as autonomous individuals in the manner of liberal political philosophy violates our self-understanding, as feminist philosophers have emphasized (e.g., Mansbridge and Moller Okin, 2017). Similarly, we can form close relationships with our colleagues at work or in joint projects that influence our understanding of who we are. This inclusive understanding of identity allows us to understand why we readily experience pride when a close family member or friend or a colleague is successful in his or her life. However, instead of feeling proud of the particular achievement of another person, it seems that we typically feel proud of the *improvement in social status* that one group member’s success grants to the entire group and its members. Indeed, it appears to be a social fact that family members of successful people benefit from the success of one of them in the form of a status increase or rise of the family among its peers. Similarly, the success of one’s close friend or colleague may improve one’s social status, although this rise typically receives less social recognition from others as our friendships and collegial relationships are less public than our family relationships, and therefore, the success of a friend or colleague may psychologically be a less reliable source of group-based pride than the success of a family member.

If group-based pride in families and close relationships relates to the rise of social status on the basis of another group member’s success, we can ask whether this is an appropriate object of group-based pride. If people are esteemed for their family backgrounds and achievements associated with them, and this social esteem increases our self-worth, then group-based pride of this kind is genuine. These considerations are particularly relevant in collectivist honor cultures where group status is the main concern of pride and its maintenance. However, high social status is valued everywhere, not merely in honor cultures, and it can be achieved either by our own achievements or by those of family members or people with whom we affiliate.

Even so, social status as such does not seem to be a plausible personal or even group-based ideal in light of which we can value ourselves as individuals or members of groups. Rather, personal or group-based ideals are those values whose achievement or promotion provides a reason for the social esteem of others and, hence, for the rise or maintenance of our social status. In the case of individuals, such ideals may include belonging to a venerable educational institution, or to a city’s glorious musical heritage, or of solidarity with a certain social class, to use Brady’s (2017) examples. Ideals of this kind are self-conceptions that matter to us and in light of which we regard our lives as valuable, and the social esteem that associates with these ideals manifests their value. Similarly, in honor cultures, group-based pride should be felt about the advancement or protection of an important value on which there is wide convergence within the culture and on which social status in the culture is based. Therefore, valuing social status for its own sake is normatively wrong-headed as it undermines the values upon which social status is founded—even in collectivist cultures. Accordingly, feeling proud of the rise of one’s social status, awarded by an achievement of a family member or a close friend is not rationally appropriate.

Nevertheless, the pride of parents about the success of their offspring seems to be warranted on a different ground, namely the parents’ indirect contribution to their children’s success that resembles social groups, both *I-mod*e and, especially, *we-mode*. Parents may feel appropriate group-based pride in the success of their children insofar as they have through their loving and caring nurturing contributed to the children’s success. Of course, loving and caring parents do not think about what kind of emotional rewards they can later on expect from their parenting—indeed, instrumental considerations of this kind would defeat the loving and caring quality of parenting, as well as the appropriateness of parental pride in the success of the children. Nor should parents feel proud of the particular achievements of their children. Pride for them belongs to the successful children, while the pride of parents relates to their contentment and joy about the outcome of their parenting. Indeed, these positive emotions are even more appropriate than pride for parents, and all these emotions probably are mixed in actual experiences of celebratory emotions that parents feel upon the success of their offspring (e.g., Sullivan and Strongman, 2003). This ground rarely applies to group-based pride about the success of one’s friend. But of course it is possible to support one’s friend in his or her important project to such an extent that its successful completion gives rise to a warranted group-based pride that becomes mixed with one’s joy for the friend’s success. This is even more likely in a supervision relationship between a teacher and a student where the achievements of the student may give rise to the teacher’s pride in his or her supervision. Yet even if there is little or no contribution to an achievement of a friend or a student with whom one group identifies, group-based pride can be appropriately felt on the basis of sharing the same value that the person’s achievement manifests, provided that the value is a focus of a coherent, rationally interconnected pattern in the subject’s actual and counterfactual emotions (Helm, 2001).

## Appropriateness of Size

Finally, after discussing the appropriate shape of group-based pride, there is the dimension of size in considering the rational appropriateness of this emotion. The *we-mode* vs. *I-mode* distinction is relevant here as the members of *we-mode* or *I-mode* groups who have achieved their shared goal with their collective efforts are warranted to express and experience more intense pride than those *I-mode* group members whose pride is associated with the achievements of others with whom they affiliate through a private group identification. An intense experience and expression of *I-mode* group-pride without a contribution to the group’s achievement betrays basking in the reflected glory of others. This seems to happen all the time, but what we actually see on stadiums and spectator stands are shared *I-mode* group-based emotions that, by virtue of their sharing, become more intense and mixed with other emotions than individually experienced emotions of the same type. Therefore, we should not make judgments on the intensity of *I-mode* group-based pride on the basis of those cases.

Intensity may be a problem also with parents and grandparents who boast about the achievements of their offspring to others, as this kind of flamboyant expression betrays inappropriate intensity of the emotion. Yet appropriateness in size does not depend merely on intensity but also on the particular other or others to whom the emotional expression is directed.^5^ If the target of an intense expression is the person whose achievement serves as the intentional object of group-based pride, such as one’s daughter, an intense expression may serve as a way of manifesting and reinforcing the value of the achievement to the person herself. In these cases, the value of an achievement may even be social in a way that it is partially constituted through the recognition of particular others. An example is when parents and other family members praise a child on the occasion of his or her graduation from school that is typically expressed by saying “I am/we are so proud of you.” Here group-based pride expressed toward the graduate him- or herself—even if it is expressed in a manner that suggests literal pride in the other person’s achievement—may serve as a way of manifesting and reinforcing the value of the achievement rather than as taking any credit for the other person’s achievement.

However, the situation changes if the other or others to whom an intense expression of a group-based emotion is targeted is someone other than the person whose achievement serves as the reason for the emotion, such as other parents or grandparents. Basking in children’s reflected glory seems particularly likely if the children are seen as part of the self, as is the case in some parents’ perceptions of their offspring. Brummelman and others suggest that parents with unfulfilled ambitions “may derive meaning from parenthood by vicariously resolving their unfulfilled ambitions through their children. Basking in children’s reflected glory, parents’ feelings of regret and disappointment about their own lost opportunities may gradually resolve, and make way for pride and fulfillment” (Brummelman et al., 2013, p. 2). However, these psychological benefits of parenthood should not be reaped at the expense of children who become assets in social rivalry if parents or grandparents boast about their achievements with the same or even higher intensity than if they were their own. This kind of behavior is morally repulsive because human beings should not be treated as means but as ends in themselves, to use Kant’s expression. In order to adhere to this moral principle, subjects of group-based pride should tone down the intensity of their emotion, recognizing the origin of the emotion in the actions of others who are justified in expressing the emotion more intensely.

An important qualification here is that parents and grandparents may feel more intense pride about the achievements of their offspring than the child him- or herself when the latter is not old enough to feel proud of those achievements. For instance, very young children may not understand their achievements such as learning to walk or to use the toilet as developmental milestones of which they could be proud, nor do they have a reflexive sense of the self that is a precondition for the emergence of self-conscious emotions until the third year of life (Lewis, 1995). When caretakers feel proud of these milestones of their offspring, this pride can be based on an empathetic understanding of the child’s situation from a perspective that the child him- or herself is incapable of taking at the time, as we suggested above. However, children move beyond this condition as they mature and learn to appreciate their developmental, academic, and social achievements. If parents or grandparents continue to feel more intense pride in the achievements of their adolescent or adult child than the child her- or himself, something is wrong. Social rivalry can be influencing the emotion, or the child might be perceived as a part or extension of the personal identity. Importantly, both of these motives can be internalized so deeply that one no external audience is needed for experiencing and expressing the relevant emotions.

It is important to note that this reasoning does not generalize to contexts where group-based emotions are shared with other group members. For instance, it does not apply to sports fans who celebrate the success of their favorite team in stadiums or in public viewing areas where their shared emotions serve the purpose of reinforcing the participants’ collective identity. In such situations, sharing intensifies individual group-based emotions into the kind of collective effervescence described already by Émile Durkheim (1995). The fact that other factors and motives influence the experience and expression of emotions in collective contexts renders those situations more complicated than the case of parents and close relationships. Indeed, *group-shared* emotions (Menges and Kilduff, 2015) experienced together with other group members differ from *group-based* emotions experienced alone in so many ways these types of emotions should not be conflated in the first place.^6^

## Conclusion

In this article, we have suggested that the question of appropriateness of group-based pride concerns both the shape and size—or the intentionality and intensity—of these emotions, as D’Arms and Jacobson (2000) point out. Regarding the appropriate shape of this emotion, we first distinguished between group-based pride and group-based hubris where the latter whose evaluative content differs from that of group-based pride qualified as a categorically inappropriate group-based emotion due to the arrogant, contemptuous, and other-derogating character of this emotion. We then discussed the appropriate shape of group-based pride in two importantly dissimilar group contexts: *we-mode* groups based on a collective commitment of the members to a group ethos; and *I-mode* groups based on personal group identification. While operative group members of a *we-mode* group are causally responsible for particular achievements, all group members can feel rationally appropriate pride in the achievement insofar as they have participated in decision-making on the group’s goals. In *I-mode* groups, individual members can feel appropriately proud of the achievement of their group similarly to *we-mode* groups if they have contributed to the achievement. If there is no contribution to a group achievement, group-based pride based on a private group identification can nevertheless be rationally appropriate if it manifests the person’s reduced-agency ideals (Brady, 2017) and is also part of a coherent pattern of rationally interconnected emotions, both group-based and individual, focused on the ideal (Helm, 2001). Moreover, we argued that families and close relationships cannot be modeled on either we-mode or I-mode groups, because they typically are normatively weaker than we-mode groups but both normatively and affectively stronger than I-mode groups as the reciprocal social commitments and affective ties in families and close relationships need not be mediated through a group ethos. We suggested that when we feel proud of the success of one’s family member or a close friend or colleague, we typically do not feel proud of the particular achievement of another person, but rather of the *rise of social status* that one group member’s success grants to the entire group and its members. However, valuing high social status for its own sake is normatively wrong-headed as it undermines the values upon which social status is founded. Therefore, group-based pride in the rise of one’s social status is not rationally appropriate. Even so, direct or indirect causal contribution to the success of one’s child, friend, or student may warrant group-based pride, which may be justified on the basis of shared values without causal contribution as well.

Finally, regarding the appropriate size of group-based pride, we suggest that the members of *we-mode* and *I-mode* groups who achieved their shared goal through their collective efforts are warranted to experience and express more intense pride than members of *I-mode* groups who feel proud of the achievements of others. Moreover, we suggest that the proper intensity of this emotion depends on the particular other(s) to whom the emotional expression is directed. If this is the person whose achievement is at stake, an intense expression may be a way of recognizing the value of the achievement; whereas if they are some others, an intense expression may betray using another person’s success as a means in social rivalry, which is morally objectionable. Finally, we point out that criteria of appropriate size may not apply to shared group-based pride as sharing increases the intensity of emotion by default. The interplay of group-based and collective emotion is a complex issue as groups regulate the expressive and other actions of in-group members when they act on behalf of the group. Here we can see a commitment and “fusion” of personal and group identity that resembles the intensity and intimacy of family groups. Therefore, the issue of rational appropriateness of group-based pride in actual group situations calls for further analysis in which we cannot engage here.

## Author Contributions

MS was responsible for outlining the argumentative structure of the manuscript, whereas GS commented the manuscript throughout and provided material from social psychological research on group-based pride and group-based hubris which are his core expertise. Both authors contributed to the article and approved the submitted version.

## Conflict of Interest

The authors declare that the research was conducted in the absence of any commercial or financial relationships that could be construed as a potential conflict of interest.

## Publisher’s Note

All claims expressed in this article are solely those of the authors and do not necessarily represent those of their affiliated organizations, or those of the publisher, the editors and the reviewers. Any product that may be evaluated in this article, or claim that may be made by its manufacturer, is not guaranteed or endorsed by the publisher.

## Footnotes

1 D’Arms and Jacobson (2000) identify a problem with different types of reasons that are relevant for the appropriateness of emotions. The problem is that insofar as we are incapable of discriminating between relevant and irrelevant reasons, we conflate these reasons together. Prudential, strategic, moral, and all-in reasons may repudiate an emotion even though other reasons render it appropriate. We may imagine situations, for instance, in funerals and other formal ceremonies, where it would be rude and imprudent and therefore all-in unreasonable to be amused by a particular event, even though the event is actually funny. D’Arms and Jacobson suggest that there are reasons that refer to the kind of appropriateness that associate with the fittingness of particular objects of emotion with the formal objects of the relevant emotion type. They call these reasons “fitting reasons,” and they are the kind of reasons we are interested in this paper. The criterion of rational appropriateness is also sensitive to the reasons that are available to the subject of emotion. Group-based pride may be an appropriate emotion for a group member who has received news about the success of his or her group. But it is not rationally appropriate for the group member until this news has reached him or her even if the emotion is fitting as soon as the success has occurred. Indeed, it is a logical criterion of pride that it requires a belief about a personal or group-based achievement, for no other epistemic attitude besides belief can ground pride. Of course, we can anticipate or imagine an achievement and thereby have a foretaste of this emotion. But in order to robustly feel proud, one needs to have the relevant belief about a realized achievement (see Gordon, 1987).

2 We realize that it is difficult to adjudicate whether particular collectivist cultures are examples of full-blown or weak we-mode groups that are theoretical models. However, both types of we-mode groups allow the members to understand themselves in terms of their group membership, which has implications on their concerns and emotions. Thus, the members understand their identity in terms of the constitutive concerns of the group that they believe to be shared by others in the group. Accordingly, the concerns shared as group members are “our concerns,” and events that advance or damage those concerns affect individual group members who experience group-based emotions in response to such events.

3 Rawls (1971) presents two principles of justice. The first principle is *the greatest equal liberty principle* whereas the second principle divides into *the difference principle* and *the equal opportunity principle* that are implemented in the opposite order, beginning from the equal opportunity principle, followed by the difference principle.

4 A complicated example could be a case in which a parent feels proud of a child because the child has achieved something significant without the parent’s help, or in spite of obstacles that the parent has set in the child’s way in order to develop his or her character. It seems important for parental pride in these cases, however, that the child whose merits are an object of parental pride is willing to let his or her demanding parents share in his or her success. The child’s willingness to share his or her pride with the parents suggests that the parents’ upbringing is perceived as ultimately beneficial and benign even if it was painful at the time. By contrast, if the child is offended by a parent who feels proud of his or her success, this suggests that the parental pride may be inappropriate. Unfortunately, demanding and psychologically controlling parents are often capable of convincing their children about the justification of their pedagogical measures so that the children do not protest against the parents nor against their pride in the children’s achievements. Therefore, the appropriateness of parental pride remains difficult to adjudicate in these empirical cases. However, there is anecdotal evidence of the mixed feelings that such children as successful adults have about their upbringing by their parents (Alessandri and Lewis, 1996).

5 We thank Alba Montes Sánchez for directing our attention to this distinction.

6 Menges and Kilduff (2015) argue that “the distinction between group-shared emotions and group-based emotions is useful as it separates collective, synchronous, interactive experiences of group emotions from individual, asynchronous, non-interactive emotional experiences based on group membership. Whereas group-shared emotions imply both similarity of feeling and the sharing of those feelings among group members, group-based emotions imply only similarity in feelings among group members” (p. 851).
